# New Feedstock System for Fused Filament Fabrication of Sintered Alumina Parts

**DOI:** 10.3390/ma13194461

**Published:** 2020-10-08

**Authors:** Dorit Nötzel, Thomas Hanemann

**Affiliations:** 1 Karlsruhe Institute of Technology, Institute for Applied Materials, Hermann-von-Helmholtz-Platz 1, 76344 Eggenstein-Leopoldshafen, Germany; dorit.noetzel@kit.edu; 2 Department of Microsystems Engineering, University Freiburg, Georges-Köhler-Allee 101, 79110 Freiburg, Germany

**Keywords:** fused filament fabrication, 3D Printing, FFF/FDM, composites, ceramics, alumina

## Abstract

Only a few 3D-printing techniques are able to process ceramic materials and exploit successfully the capabilities of additive manufacturing of sintered ceramic parts. In this work, a new two component binder system, consisting of polyethyleneglycol and polyvinylbutyral, as well stearic acid as surfactant, was filled with submicron sized alumina up to 55 vol.% and used in fused filament fabrication (FFF) for the first time. The whole process chain, as established in powder injection molding of ceramic parts, starting with material selection, compounding, measurement of shear rate and temperature dependent flow behavior, filament fabrication, as well as FFF printing. A combination of solvent pre-debinding with thermal debinding and sintering at a reduced maximum temperature due to the submicron sized alumina and the related enhanced sinter activity, enabled the realization of alumina parts with complex shape and sinter densities around 98 % Th. Finally the overall shrinkage of the printed parts were compared with similar ones obtained by micro ceramic injection molding.

## 1. Introduction

Over recent years, a huge variety of additive manufacturing (3D printing) methods have been developed, starting with polymer materials and extended nowadays to ceramics, metals, composites, or other advanced functional materials. Actually, the inclusion of time as a fourth dimension has been started and is denoted as 4D Printing [1,2,3,4,5,6]. 3D printing allows the realization of “impossible” parts with particular geometrical features, which cannot be produced applying established fabrication methods. Related to material processing, 3D printing can be distinguished in seven categories, namely material extrusion, material jetting, binder jetting, powder bed fusion, direct energy deposition, vat polymerization, and sheet lamination [7]. Originally, most of these were used for the processing of polymer-based materials, like UV-curable reactive resins in case of vat polymerization (Stereolithography, SLA) or thermoplastics in the case of extrusion (Fused Filament Fabrication, FFF). Comprehensive material research in close cooperation with machine technology development enabled, that SLA and FFF can be used recently for the realization of functional composites e.g., with piezoelectric, conductive, ferroelectric, or magnetic properties [8,9,10,11,12]. Whilst metal parts are mainly printed directly by the different variants of Selective Laser Sintering (SLS), Selective Laser Melting (SLM), or quite recently by Electron Beam Melting (EBM) [13,14,15,16], ceramic parts can be printed via SLA and FFF using highly filled low viscous resins (SLA) or molten thermoplastics (FFF). In the case of SLA, this technology has been commercialized (Lithoz GmbH, Vienna, Austria, www.lithoz.com), recent research can be found in [17,18,19,20,21,22]. The fabrication of ceramic or metallic parts by FFF has the major advantage that experience derived from powder injection molding (PIM) helps to develop highly filled feedstocks (ceramic: >45 vol.%, metal: >60 vol.% solid load) with a similar binder composition [23,24]. Considering only ceramics, up to now mostly sintered alumina or mullite [23,24,25,26], zirconia [27,28,29], or silicon nitride [30] parts have been realized, typical binder components are paraffin wax, (modified) polyolefines, or thermoplastic elastomers. Fatty-acid derivatives, like stearic acid, or commercial additives with proprietary composition, are widely established as surfactants or plasticizers [23,24,25,27,28,30,31]. Like feedstocks in ceramic injection molding, two component binder mixtures are often applied, one low molecular mass material allows a low melt viscosity at moderate temperatures and one high molecular mass fraction (backbone polymer) delivers a certain mechanical stability at lower temperatures after shaping. The low molecular mass compound can be removed during an organic solvent pre-debinding step after printing, e.g., in hexane [23,24] or cyclohexane [28]. In micro ceramic injection molding, new binder systems avoiding the necessity to use organic solvents for pre-debinding have been developed. Polyvinylbutyral (PVB) or polymethylmethacrylate (PMMA) as backbone polymer and polyethyleneglycol (PEG) as low molecular mass component were successfully tested with ceramic and metal fillers [32,33,34], even the usage of by FFF or PolyJet^®^ (3D Inkjet) printed mold inserts was possible [35]. After replication and during debinding, the low molecular binder component PEG can be dissolved in cold water instead of the highly flammable hexane at 50 °C.

This work describes the first-time implementation of the PEG/PVB binder system as ceramic filler carrier in ceramic FFF 3D printing. The realization and structural characterization of complex alumina parts will be presented in detail.

## 2. Materials and Methods

Regarding the realization of dense ceramic parts via FFF a process chain analogous to injection molding was developed. This was successfully demonstrated earlier applying a binder system consisting of wax and polyethylene [23,24] covering the following steps:

1. Material (filler, binder, and surfactant) selection;
2. Compounding and rheological characterization;
3. Filament extrusion;
4. Feedstock printing; and
5. Thermal post-processing (debinding, sintering)

For each step, a comprehensive material characterization has to be investigated with respect to develop a robust process chain enabling dense and warpage-free ceramic parts.

### 2.1. Material Selection

As in previous work—FFF and micro injection molding [23,36]—a submicron sized alumina (Al_2_O_3_^,^ TM-DAR, Taimai Chemicals, Tokyo, Japan) was selected as ceramic because of its huge sinter activity enabling density values better than 99% at moderate sinter temperatures (1400 °C) [37]. The measured average particle size *d*_50_ is around 0.1 µm, the specific surface value, measured via the BET (Brunauer-Emmett-Teller) method, is 11.8 m²/g [23]. In contrast to the established binder system, wax/polyethylene in powder injection molding used for alumina, zirconia, tungsten, and stainless steel [38,39], a partially water-soluble binder will be applied. Polyvinylbutyral (PVB, Mowital B30H, Kuraray Europe GmbH, Hattersheim/Main, Germany) was used as backbone polymer (glass transition temperature 68 °C, melting temperature 135–170 °C) delivering a certain mechanical stability at lower temperatures. Polyethyleneglycol (PEG 4000, Roth GmbH, Karlsruhe, Germany) was selected as low molecular weight plasticizer (softening temperature 53–58 °C) enabling a reduced viscosity during compounding. To achieve a reliable coupling between the hydrophilic ceramic and the hydrophobic polymer matrix, stearic acid (SA, Roth GmbH, Karlsruhe, Germany) was chosen as surfactant guaranteeing a low feedstock viscosity and good homogeneity in the molten state. The SA concentration was set to 3.3 mg/m² referred to the alumina’s specific surface area guaranteeing a complete ceramic particle surface coverage [36]. The surface profile (2D and line scans) were measured applying a white light interferometer (Micro Prof^®^ 100, Fries Research Technology, Bergisch Gladbach, Germany).

### 2.2. Compounding and Rheological Characterization

All feedstocks have been prepared in a mixer-kneader compounder (W50-EHT, Brabender, Duisburg, Germany) which allows for torque recording during mixing. The compounding temperature was set to 110 °C, the mixing time to 60 min guaranteeing the formation of a homogeneous feedstock. To ensure an operator independent processing, a fixed sequence of the addition of the individual components was defined: First, approximately 20% of the alumina was filled in the mixing chamber (volume around 45 mL). Second, the surfactant was added enabling an improved surface coverage and prior to the polar binder parts, which also can bind via physisorption to the ceramic. Third, the premixed PEG/PVB was adjoined and the remaining filler was placed in the mixing chamber finally. A typical torque vs. time compounding curve can be seen in [23] for a similar highly filled composite applying a wax-polyethylene binder system. The shear rate and temperature dependent flow properties have been measured by high pressure viscosimetry using a Rheograph 25 (Göttfert, Buchen, Germany). A temperature range from 140–160 °C was selected for the measurements, the shear rate was varied between 1 and 8000 s^–1^. The solid loadings were set to 50 and 55 vol.% which are equivalent to 77.4 and 80.9 wt.%. Similar feedstocks with huge alumina amount (up to 58 vol.%) were previously used in powder injection molding [34,35].

### 2.3. Filament Extrusion and Printing via FFF

All feedstocks were dried in a vacuum oven (Heraeus, Hanau, Germany) over night at 44 °C to remove adsorbed water, which is favorable due to the very hydrophilic binder properties. Finally, they were extruded to filaments applying a filament extruder (Noztek pro HT, Noztek, Shoreham, UK) at 110 °C (50 vol.%) and 115 °C (55 vol.%), respectively. The selected nozzle diameter was 2.8 mm. The resulting filaments were too brittle for winding; therefore, they were cut every 50 cm for direct feeding.

The filaments were used for 3D printing applying a modified FFF desktop printer (X350 pro, German RepRap, Feldkirchen, Germany). The conducted modifications mainly affect changes at the print head e.g., using a Titan extruder with a gear ratio of 3:1 allowing a more precise filament conveying [23]. The temperature of the polypropylene printing plate was set to 60 °C; the printing temperature was 165 °C with a printing speed of 10 mm/s. The nozzle was made from hardened steel due to the pronounced abrasion of the alumina feedstock; the nozzle´s inner diameter was 0.25 mm enabling a printed layer thickness of around 0.1 mm.

### 2.4. Debinding and Sintering

After printing and prior to sintering, all-organic moieties have to be removed by debinding either in a single thermal treatment or in a combination of solvent-assisted liquid removal (pre-debinding) of the low molecular PEG. The latter method was established for the fabrication of small and dense alumina and zirconia parts [33]. As shown there, a combination of solvent pre-debinding in water and thermal debinding was applied for removing the low molecular compound PEG. For debinding and sintering two chamber ovens (HT6/28 and RHF17/3, both from Carbolite, Neuhausen, Germany), were applied. The Archimedes method was used for the measurement of the sample densities.

## 3. Results and Discussion

### 3.1. Material Selection

**Figure 1:**
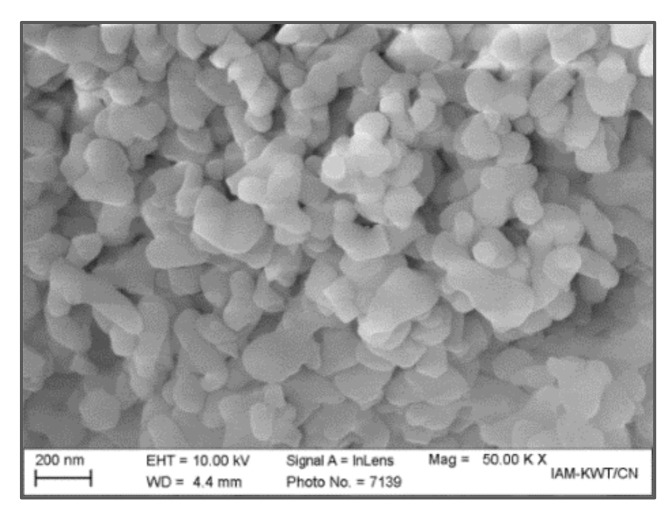
SEM-image of used nanosized alumina TM-DAR.

The relevant properties of the used alumina particles have been reported earlier, the morphology obtained by SEM investigations shows mostly spherical nanosized primary particles, which are highly agglomerated (Figure 1) [23]. The measured specific surface area is relevant for the calculation of the surfactant amount needed; here an SA concentration of 3.3 mg/m² related to the particle surface was selected guaranteeing a complete coverage of the alumina’s surface enabling the formation of a homogeneous feedstock after compounding. The calculation of the feedstock composition is as follows, related to 100 g equivalent to 39.85 cm³ feedstock: The solid load, for example 50 vol.%, defines the amount of SA due the above mentioned calculation ruled by the alumina´s total surface area. Half of the remaining volume amount, here 50 vol.%, is attributed to PEG being the low viscous binder component. The remaining fraction to 100 % consists of the calculated amount of SA and PVB. As an example, 50 vol.% alumina equals 77.5 wt.% equals 77.5 g equals 915 m² surface area in total. Under consideration of the given SA concentration, 3.3 cm³ (3.0 g) surfactant is necessary. As described, 9.96 cm³ (12.2 g) equals the PEG amount, only 6.68 cm³ (7.3 g) remains for PVB (all numbers are rounded). If necessary, this composition can be slightly modified if a lower viscous feedstock is needed (increasing PEG amount) or higher green stability (higher PVB amount) is targeted [34].

### 3.2. Compounding and Rheological Characterization

**Figure 2:**
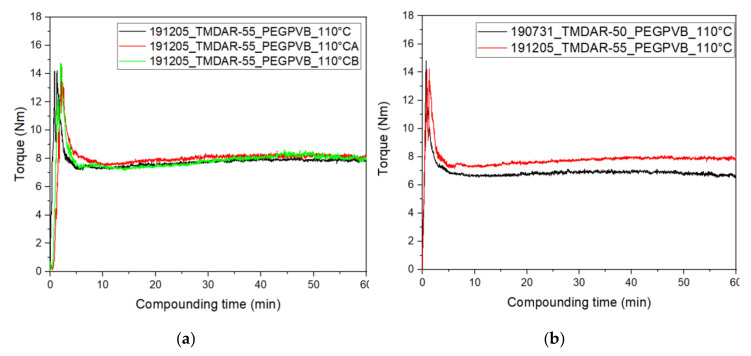
Compounding diagrams using the torque recording mixer/kneader of alumina in polyethyleneglycol (PEG)/polyvinylbutyral (PVB) (T = 110 °C, 30 rpm); (**a**) Record of three different measurements with identical feedstocks (55 vol.%); (**b**) Torque comparison at different solid loadings.

The use of an in-line torque recording mixer-kneader allows during compounding a validation of the homogeneity of the feedstock, derived from the fluctuation of the measured torque signal and the resulting final equilibrium value. The latter one gives a strong evidence for successful injection molding or here FFF-printing as a rule-of-thumb the equilibrium value should be below 10 Nm for further processing. The compounding behavior of the investigated PEG/PVB/SA/alumina mixtures is shown in Figure 2. Three identical compositions were prepared showing the excellent repeatability of the compounding process even at a high solid load of 55 vol.% (Figure 2a).

Following the time proceeding torque values after 10 min almost equilibrium is reached, i.e., most agglomerates are destroyed and all particles are wetted by the surfactant SA. Figure 2b shows in addition the influence of the alumina load on the compounding process, the load reduction down to 50 vol.% lowers the equilibrium torque value from approximately 8 Nm down to 6.7 Nm under identical processing conditions. A mixing temperature of 110 °C was selected due to the pronounced thermal decomposition of PEG at elevated temperatures, for better comparison one compounding process was performed at 125 °C with an equilibrium torque value of 4.7 Nm (50 vol.% alumina, not shown here). A comparison with similar feedstock compositions delivers an interesting behavior: Wax/PE feedstocks with the same alumina and a solid load of 50 vol.% resulted in a final torque value around 5 Nm (~125 °C), which is almost identical to the PEG/PVB based feedstock [23]. Applying the same binder composition, but a different alumina with larger average particle size and slight smaller specific surface area (Martoxid MR70, *d*_50_: 0.5–0.8 µm, *A*_BET_ = 8.6 m²/g), the equilibrium torque was approximately around 5 Nm (50 vol.%) and 7 Nm (55 vol.%), measured at 125 °C [34]. As a result, under the given compounding conditions, the three different feedstocks behave quite similar demonstrating that the solid load is significantly below the critical filler load going along with a tremendous torque and viscosity increase.

**Figure 3:**
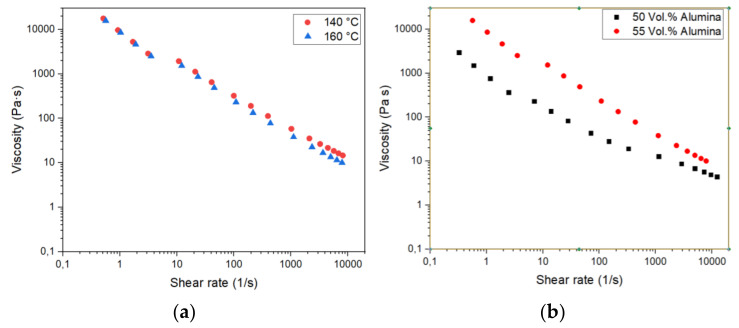
Rheological investigation on the alumina containing feedstocks: (**a**) Viscosity at two different temperatures (load: 55 vol.%) and (**b**) Two different solid loadings (temperature: 160 °C).

High-pressure capillary rheometry allows for a more precise description of the feedstock flow behavior in the molten state, especially for the change of the viscosity with shear rate, denoted as pseudo plasticity, and solid load. Figure 3 describes the dependency of the melt viscosity as a function of shear rate, temperature, and solid load. An increase of the measuring temperature from 140 to 160 °C cause only a small viscosity drop at higher shear rates, which is relevant for powder injection molding with its huge melt injection speed, but not significant for FFF with low shear rates during filament deposition (Figure 3a). In contrast, there is a pronounced impact of the solid load on the viscosity, especially at lower shear rates. In FFF, typical shear rates are in the range of 100 s^–1^, a rough estimation from Figure 3b yields a significant higher viscosity for the feedstock with 55 vol.% load (approximately 230 Pa⋅s) in comparison to the one with 50 vol.% (approx. 35 Pa⋅s). The analogous wax/PE feedstock shows under identical conditions higher viscosity values around 270 Pa⋅s (50 vol.%) and 350 Pa⋅s (55 vol.%), both measured at a shear rate of 100 s^–1^ as well [23]. In the case of the identical binder but different alumina, a similar viscosity at 50 vol.% (30 Pa⋅s) was found, but at higher solid loadings (55 vol.%) the viscosity is increased (480 Pa⋅s) also [34]. Hence, it is recommended to use feedstocks with a moderate alumina load for FFF-printing allowing for parts with reduced number of defects.

### 3.3. Filament Extrusion and Printing via FFF

**Figure 4:**
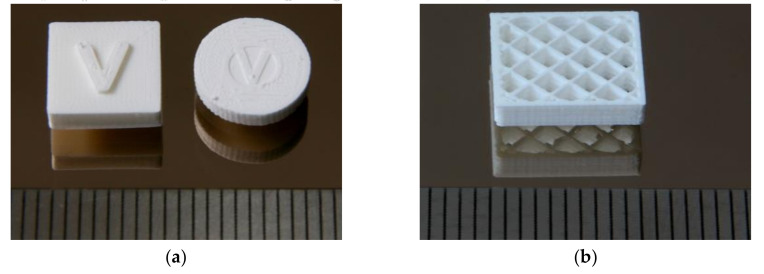
Printed test structures. (**a**) Massive body (left: cuboid, right: disc) and (**b**) Open grid.

As described in Section 2.3, the different highly filled feedstocks were extruded to filaments. These were too brittle for winding, hence, they were placed directly in the printer´s extruder hopper for feeding. Feedstock with 55 vol.% were too brittle for extruding and printing, therefore all presented parts contain only 50 vol.% alumina load. Specimen and different demonstrators were printed applying the above listed (Section 2.3) base printing parameters. Figure 4 shows two different types of printed test structures—massive type with complete infill (**a**) and fragile grids (**b**)— both typically denoted in ceramic processing as greenbodies. In the first case (Figure 4a), the printed V-structure indicates the printing direction. The greenbodies do not show any delamination and warpage after printing. Table 1 summarizes all relevant parameters for the new PEG/PVB based feedstock system in comparison to the established wax/PE-system reported in [23,24]. Two major information can be deduced from this table, first the printing parameters are quite similar, and second a reduced solid load in case of the PEG/PVP-mixture is necessary for successful printing. This comes directly from the viscosity measurements described earlier and causes a higher shrinkage during thermal post-processing due to the larger organic binder moiety.

**Figure 5:**
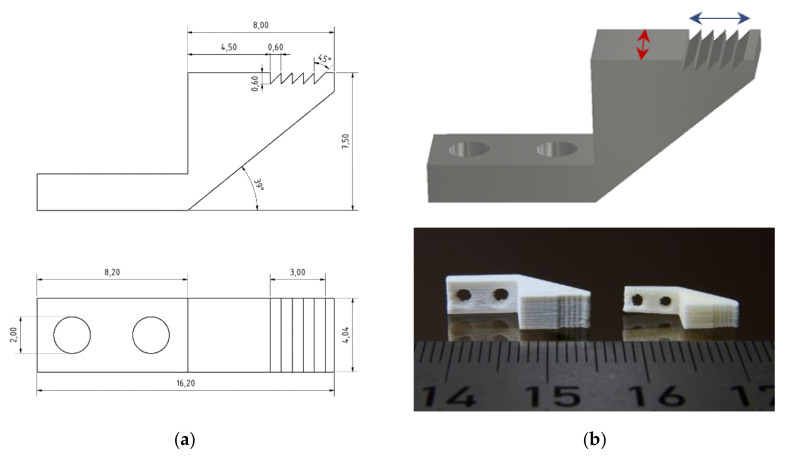
Clamping test structure: (**a**) 2D CAD-drawing and (**b**) Upper image: 3D CAD-drawing indicating scan directions, lower image: printed greenbody and sintered part.

**Figure 6:**
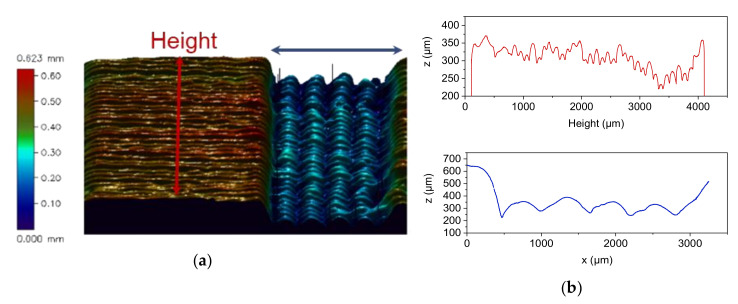
Surface topography analysis via white light interferometry: (**a**) 3D-surface scan and (**b**) upper image: profile of the structure´s sidewall quality along the red arrow, lower image: profile of the serrated structure along the blue arrow.

A more ambitious test structure was investigated estimating the achievable structural design quality and accuracy after printing. Figure 5a shows the CAD-drawing of the used clamping test structure. Beyond the standard geometric features (outer x,y,z-dimensions), the asymmetric serrated profile is challenging for FFF-printing. Figure 5b, upper image, indicates the surface line scan directions (red and blue arrows) shown in Figure 6. Figure 5b, lower image, shows the printed greenbody and the related sintered alumina part. Figure 6a gives an overview about the surface topography measured of FFF-printed part, the individual printed layers can be easily seen, which is typical, especially in the case of FFF-printing, even if the smallest accessible printing nozzle is used. A closer look to the achievable surface quality via line scans is presented in Figure 6b. Figure 6b, upper image, delivers a scan along the red line in Figure 6a showing a repeatability of the sidewall position around ±75 µm. The structure was placed on the printing platform with the largest surface area. The scan along the red line measures the layer-by-layer deviation during printing from the first printed layer to the last one (Figure 6b, upper image). The scan along the blue line represents the profile of one printed layer along the serrated structure (Figure 6b, lower image). It is obvious, that the pristine asymmetric shape of the serrated structure was not printed in a correct way.

### 3.4. Debinding and Sintering

With respect to dense and warpage-free ceramic parts, a solvent pre-debinding step for 24 h in water (25 °C) prior to thermal debinding and subsequent sintering was necessary. The following thermal treatment was performed very slowly not to activate any delamination between the individual printed layers due to evolved gaseous binder decomposition products (Table 2). As known from previous work, thermal debinding is completed around 500 °C [33]. With respect to the enhanced sinter activity of the TM-DAR alumina, a dilatometric investigation of a pressed specimen were done up to a temperature of 1600 °C [23] showing, that sintering started around 1000 °C and is finished at 1400 °C when contraction due to sinter shrinkage ended. The fragile parts were sintered at a maximum temperature of 1400 °C following a simple thermal program (Table 3). The achieved part density measured by the Archimedes method is approximately 98% of the theoretical density. This is slightly below earlier values achieved, e.g., by reaction molding (99.2% Th.) [36] or injection molding (99.7% Th.) and can be attributed to voids generated during filament deposition during FFF-printing [23,24,39]. In case of the wax/PE-system a further load increase to 55 vol.% yielded a density increase up to 99.6% Th. [24].

**Figure 7:**
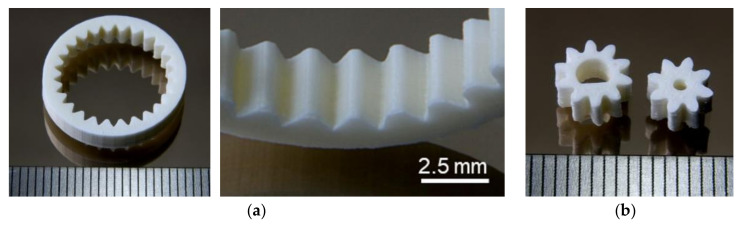
Sintered alumina parts: (**a**) Complete ring gear and structure details and (**b**) gear wheels.

In the following, more complex sintered test structures will be presented demonstrating the applicability of the new feedstock composition for the realization of alumina parts by FFF. Figure 7a shows one ring gear and structure details of the inner teeth, Figure 7b two examples for different gear wheels. In both cases, a good surface quality can be seen.

### 3.5. Process Chain Evaluation

The previous reported wax/polyethylene feedstock and resulting achievable part quality as well as process robustness serve as benchmark for the new PEG/PVB system. Table 4 compares the process chain for ceramic part fabrication for the new feedstock with the previous reported one [23,24]. All individual process steps can be done in comparable quality, only the maximum printable alumina load is reduced in case of the PEG/PVB mixture.

In contrast to the wax/PE binder system the solvent-based debinding step was done in cold water instead of hot hexane (50 °C), which avoids the usage of the non-healthy and flammable hexane and simplifies the process chain significantly. Following the presented results, the new PEG/PVB binder system is a suitable alternative to the established ones.

### 3.6. Comparison with Injection Molding

A second benchmark is the comparison of FFF with injection molding applying an identical feedstock (composition, solid load, here 50 vol.%). Due to technical reasons (number of produced parts for better statistics), the FFF-printed cuboid structure was compared with the injected molded (Battenfeld Microsystem 50, Kottingbrunn, Austria) disc structure. Both parts possess a similar thickness and volume. As expected and described in Section 3.2, injection molding with its high injection speed and pressure allows for higher ceramic densities around 100% of the theoretical density value (Table 5). The almost pressure-less FFF with the printing method related incorporation of voids during filament deposition first within the printed layer and second between the printed layers causes reduced density values around 97% Th., which are quite acceptable. Quite recently FFF-printing of a comparable system (50 vol.% alumina), ethylene vinyl acetate (EVA) as binder, SA as surfactant) allowed for similar sinter densities around (sinter temperature 1600 °C) 98%–99% Th. [25]. The listed shrinkage values (Table 5) must be explained in detail: The given deviation values do not represent the standard deviation, but the absolute deviation from the arithmetic mean value. In most of the cases, the values are identical, but in the case of the injection molded specimen a pronounced higher shrinkage value and deviation for the z-axis was observed. This influences the calculated average shrinkage value in an asymmetric manner.

## 4. Conclusions and Outlook

A new two-component polar binder system, consisting of the low molecular weight polyethyleneglycol, which acts as plasticizer at higher temperatures, and polyvinylbutyral as backbone polymer, which allows for a certain mechanical stability in the green state after shaping, was used in sintered alumina part fabrication applying the FFF 3D printing method. As being established in ceramic injection molding, only small feedstock recipe adaptions with respect to the special requirements for FFF were necessary. Feedstocks with an alumina load up to 55 vol.% could be compounded, but due to the large viscosity at printing temperature and brittle appearance a reduction down to 50 vol.% load was necessary for successful printing. After liquid pre-debinding and subsequent thermal post-treatment (debinding and sintering), dense and warpage-free alumina parts could be realized. It was possible to print and sinter, in addition to simple test specimen, even complex alumina parts like small gear wheels and gripper structures with challenging asymmetric structural details. Best achieved sinter densities were in the range of 98% of the theoretical value, which is quite comparable with data obtained from micro ceramic injection molding. The comparison of printed with similar parts, fabricated by micro ceramic injection molding, showed with respect to dimensional accuracy after printing no significant difference. Future work should consider alternative surfactants and the use of a PEG with a lower molecular weight for an enhanced plasticizing effect and a further increase of the accessible solid load reducing sinter shrinkage.

## Figures and Tables

**Table 1:** Fused filament fabrication (FFF) Printing parameters for the new feedstock system.

Item	PEG/PVP	Reference Wax/PE
Solid	Al_2_O_3_	Al_2_O_3_
Solid load (vol.%)	50	55
Extrusion temperature (°C)	<170	<170
Printing speed (mm/s)	10	10
Platform temperature (°C)	60	70
Used nozzle diameter (mm)	0.25	0.25
Printing quality	Very good	Very good

**Table 2:** Thermal debinding parameters.

Step/Temperature (°C)	Rate (°C/min)	Dwell Time @ Temperature (min)
25–120	0.2	120
180	0.2	120
250	0.2	120
500	0.2	60
500–25	n.a.	n.a.

**Table 3:** Sinter program.

Step/Temperature (°C)	Rate (°C/min)	Dwell Time @ Temperature (min)
25–1400	3	360
1400–25	10	25

**Table 4:** Comparison two different feedstocks: overall process chain evaluation.

Item	PEG/PVP ^1^	Reference Wax/PE ^1^ [23,24]
Ceramic	Al_2_O_3_	Al_2_O_3_
Compounding	✓	✓
Filament extrusion	✓	✓
FFF printing	✓	✓
Max. printable solid load (Vol.%)	50	55
Debinding	✓	✓
Sintering	✓	✓
Max. ceramic part density (% Th.)	97.1 ± 1.4	99.4 (50 vol.%), 99.6 (55 vol.%) [24]
Average shrinkage	20.75% ± 0.8%	20.4% ± 1.4% (50 vol.%); 18.0% ± 1.6% (55 vol.%) [24]

^1^✓ means process successfully established with good and reproducible quality.

**Table 5:** Comparison FFF-printed parts with injection molded ones.

Item	FFF-Printed	Injection Molded
Replicated part	Cuboid	Disc
Number of considered parts	10	10
Theoretical alumina density (g/cm³)	3.97	3.97
Average sinter density (% Th.)	97.1 ± 1.4	100
Maximum part density (% Th.)	98.5	100
Shrinkage x-axis	21.0% ± 0.5%	20.0% ± 0.3%
Shrinkage y-axis	20.8% ± 0.5%	20.1% ± 0.2%.
Shrinkage z-axis	20.5% ± 0.8%	21.7% ± 1.1%
Average shrinkage	20.75% ± 0.8%	20.6% + 2.1%−0.9%
